# Kinetic models for epidemic dynamics with social heterogeneity

**DOI:** 10.1007/s00285-021-01630-1

**Published:** 2021-06-26

**Authors:** G. Dimarco, B. Perthame, G. Toscani, M. Zanella

**Affiliations:** 1 Mathematics and Computer Science Department, University of Ferrara, Ferrara, Italy; 2 Sorbonne Université, CNRS, Université de Paris, Inria Laboratoire Jacques-Louis Lions, 75005 Paris, France; 3 Mathematics Department, University of Pavia, Pavia, Italy

**Keywords:** 92D30, 92D25, 35Q84, 35Q92

## Abstract

We introduce a mathematical description of the impact of the number of daily contacts in the spread of infectious diseases by integrating an epidemiological dynamics with a kinetic modeling of population-based contacts. The kinetic description leads to study the evolution over time of Boltzmann-type equations describing the number densities of social contacts of susceptible, infected and recovered individuals, whose proportions are driven by a classical SIR-type compartmental model in epidemiology. Explicit calculations show that the spread of the disease is closely related to moments of the contact distribution. Furthermore, the kinetic model allows to clarify how a selective control can be assumed to achieve a minimal lockdown strategy by only reducing individuals undergoing a very large number of daily contacts. We conduct numerical simulations which confirm the ability of the model to describe different phenomena characteristic of the rapid spread of an epidemic. Motivated by the COVID-19 pandemic, a last part is dedicated to fit numerical solutions of the proposed model with infection data coming from different European countries.

## Introduction

The SARS-CoV-2 pandemic led in many countries to heavy lockdown measures assumed by the governments with the aim to control and limit its spreading. In this context, an essential role is played by the mathematical modeling of infectious diseases since it allows direct validation with real data, unlike other classical phenomenological approaches. This consequently permits evaluation of control and prevention strategies by comparing their cost with effectiveness and to give support to public health decisions (Ferguson 2006; Riley 2003). On this subject, most of the models present in literature make assumptions on transmission parameters (Brauer et al. 2019; Diekmann and Heesterbeek 2000) which are considered the only responsible of the spread of the infection. However, special attention was recently paid by the scientific community to the role and the estimate of the distribution of contacts between individuals as also a relevant cause of the potential pathogen transmission (cf. (Béraud 2015; Dolbeault and Turinici 2020; Fumanelli 2012; Mossong 2008) and the references therein).

In this direction, the results reported in (Béraud 2015) can be of great help when designing partial lockdown strategies. In fact, an optimal control of the pathogen transmission of the epidemic could be achieved through a direct limitation of the number of daily contacts among people. On this subject, the detailed analysis performed in (Béraud 2015) put into evidence that the number of social contacts in the population is in general well-fitted by a Gamma distribution, even if this distribution is not uniform with respect to age, sex and wealth. Gamma distributions belong to the wide class of generalized Gamma distributions (Lienhard and Meyer 1967; Stacy 1962), which have been recently connected to the statistical study of social phenomena (Kehoe 2012; Rehm 2010), and fruitfully described as steady states of kinetic models aiming to describe the formation of these profiles in consequence of repeated elementary interactions (Dimarco and Toscani 2019; Toscani 2020).

Starting from the above consideration and inspired by the recent development concerning kinetic models describing human behavior (Dimarco and Toscani 2019; Toscani 2020), in this paper we develop a mathematical framework to connect the distribution of social contacts with the spreading of a disease in a multiagent system. The result is obtained by integrating an epidemiological dynamics given by a classical compartmental model (Brauer et al. 2019; Diekmann and Heesterbeek 2000; Hethcote 2000) with a statistical part based on kinetic equations determining the formation of social contacts.

The study of the kinetic compartmental system allows to compare the results with that obtained by studying the spread of the epidemic by means of social networks, where contacts between individuals in a given population can be captured by assuming that nodes represent individuals and the edges represent the connections between them (Hernandez-Vargas et al. 2019). In connection with this subject, by following a similar approach it was possible to understand how diseases spread in highly heterogeneous social networks (Barthélemy et al. 2005). Also, it was recently shown that a strategic social network-based reduction of contact strongly enhances the effectiveness of social distancing measures while keeping risks lower (Block 2020), and that overdispersed diseases such as COVID-19 are very sensitive to social network size and clustering (Nielsen et al. 2021).

In this paper we concentrate on the classical SIR dynamics. However, we stress that the ideas here described are clearly not reduced to this model which can be intended as an example. Instead, the methodology here discussed can be extended to incorporate more realistic epidemiological dynamics, like for instance the classical endemic models discussed in (Brauer et al. 2019; Diekmann and Heesterbeek 2000; Hethcote 2000). In particular, the extension of the present approach to age-dependent compartmental models could be of great interest to produce realistic scenarios.

Other aspects, which certainly have a stronger impact on how a virus spreads, are related to the presence of asymptomatic individuals (Gaeta 2021) as well as to a time delay between contacts and outbreak of the disease (Cooke et al. 1999) which helps in the diffusion of the illness. These possible modeling improvements are the subject of future investigations and they will not be treated in this work. In fact, we stress that the principal scope of this work is to introduce a new class of models which is capable to incorporate information on the social heterogeneity of a population which we believe to be a crucial aspect in the spread of contagious diseases. Besides these simplifying assumptions, we will show that the basic features considered and detailed in the rest of the article are sufficient in many cases to construct a new class of models which well fits with the experimental data. Precise quantitative estimates are postponed to future investigations.

An easy way to understand epidemiology models is that, given a population composed of agents, they prescribe movements of individuals between different states based on some matching functions or laws of motion. According to the classical SIR models (Hethcote 2000), agents in the system are split into three classes, the susceptible, who can contract the disease, the infected, who have already contracted the disease and can transmit it, and the recovered who are healed, immune or isolated. Inspired by the model considered in (Dimarco and Toscani 2019) for describing a social attitude and making use of classical epidemiological dynamics, we present here a model composed by a system of three kinetic equations, each one describing the time evolution of the distribution of the number of contacts for the subpopulation belonging to a given epidemiological class. These three equations are further coupled by taking into account the movements of agents from one class to the other as a consequence of the infection, with an intensity proportional to the product of the average contact frequencies, rather than the product of population fractions.

The interactions which describe the social contacts of individuals are based on few rules and can be easily adapted to take into account the behavior of agents in the different classes in presence of the infectious disease. Our joint framework is consequently based on two models which can be considered classical in the respective fields. From the side of multi-agent systems in statistical mechanics, the linear kinetic model introduced in (Dimarco and Toscani 2019; Toscani 2020) has been shown to be flexible and able to describe, with suitable modifications, different problems in which human behavior plays an essential role, like the formation of social contacts. Once the statistical distribution of social contacts has been properly identified as equilibrium density of the underlying kinetic model, this information is used to close the hierarchy of equations describing the evolution of moments (Bobylev 1988; Dimarco et al. 2020; Cercignani 1988). In this way, we obtain a coupled system of equations, identifying a new epidemiological model which takes into account at best the statistical details about the contact distribution of a population. The model connects the measure of the heterogeneity of the population, i.e. the variance of the contact distribution, with the epidemic trajectory. This is in agreement with a well-know finding in the epidemiological literature, see e.g. (Anderson and May 1985; Barthélemy et al. 2005; Bonaccorsi et al. 2020; Diekmann et al. 1990; Flaxman et al. 2020; Novozhilov 2008; Van den Driessche and Watmough 2002). A recent research showing the influence of population heterogeneity on herd immunity to COVID-19 infection is due to Britton et al. (Britton et al. 2020).

Starting from the general macroscopic model, one can fruitfully obtain from it various sub-classes of SIR-type epidemiological models characterized by non-linear incidence rates, as for instance recently considered in (Korobeinikov and Maini 2005). It is also interesting to remark that the presence of non-linearity in the incidence rate function, and in particular, the concavity condition with respect to the number of infected has been considered in (Korobeinikov and Maini 2005) as a consequence of *psychological* effects. Namely, the authors observed that in the presence of a very large number of infected, the probability for an infected to transmit the virus may further decrease because the population *tend to naturally reduce the number of contacts*. The importance of reducing at best the social contacts to countering the advance of a pandemic is a well-known and studied phenomenon (Ferguson 2006). While in normal life activity, it is commonly assumed that a large part of agents behave in a similar way, in presence of an extraordinary situation like the one due to a pandemic, it is highly reasonable to conjecture that the social behavior of individuals is strictly affected by their personal feeling in terms of safeness. Thus, in this work, we focus on the assumption that it is the degree of diffusion of the disease that changes people’s behavior in terms of social contacts, in view both of the personal perception and/or of the external government intervention. More generally, the model can be clearly extended to consider more realistic dependencies between an epidemic disease and the social contacts of individuals. However, this does not change the essential conclusions of our analysis, namely that there is a close interplay between the spread of the disease and the distribution of contacts, that the kinetic description is able to quantify. In particular, we stress the fact that we consider our approach as methodological, thus the encouraging results described in the rest of the article suggest that a similar analysis can be carried out, at the price of an increasing difficulty in computations, in more complex epidemiological models like the SIDARTHE model (Giordano 2020; Gatto 2020), to validate and improve the eventual partial lockdown strategies of the government and to suggest future measures.

The rest of the paper is organized as follows. Section 2 introduces the system of three SIR-type kinetic equations combining the dynamics of social contacts with the spread of infectious disease in a system of interacting individuals. Then, in Sect. 2.2 we show that through a suitable asymptotic procedure, the solution to the kinetic system tends towards the solution of a system of three SIR-type Fokker-Planck type equations with local equilibria of Gamma-type (Béraud 2015). Once the system of Fokker-Planck type equations has been derived, in Sect. 3, we close the system of kinetic equations around the Gamma-type equilibria to obtain a new epidemiological model in which the incidence rate depends on the number of social contacts between individuals. Last, in Sect. 4, we investigate at a numerical level the relationships between the solutions of the kinetic system of Boltzmann type, its Fokker-Planck asymptotics and the macroscopic model. These simulations confirm the ability of our approach to describe different phenomena characteristic of the trend of social contacts in situations compromised by the rapid spread of an epidemic and the consequences of various lockdown action in its evolution. A last part is dedicated to a fitting of the model with the experimental observations: first we estimate the parameters of the epidemic through the data at disposal and successively we use them in the macroscopic model showing that our approach is able to reproduce the pandemic trend.

## A kinetic approach combining social contacts and epidemic dynamics

Our goal is to build a kinetic system which suitably describes the spreading of an infectious disease under the dependence of the contagiousness parameters on the individual number of social contacts of the agents. Accordingly to classical SIR models (Hethcote 2000), the entire population is divided into three classes: susceptible, infected and recovered individuals. As already claimed the ideas here developed can be extended to more complex compartmental epidemic models.

Aiming to understand social contacts effects on the dynamics, we will not consider in the sequel the role of other sources of possible heterogeneity in the disease parameters (such as the personal susceptibility to a given disease), which could be derived from the classical epidemiological models, suitably adjusted to account for new information (Diekmann et al. 1990; Novozhilov 2008; Van den Driessche and Watmough 2002). Consequently, agents in the system are considered indistinguishable (Pareschi and Toscani 2014). This means that the state of an individual in each class at any instant of time $t\ge 0$ is completely characterized by the sole number of contacts $x \ge 0$, measured in some unit.

While *x* is a natural positive number at the individual level, without loss of generality we will consider *x* in the rest of the paper to be a nonnegative real number, $x\in {{\mathbb {R}}^+}$, at the population level. We denote then by $f_S(x,t)$, $f_I(x,t)$ and $f_R(x,t)$, the distributions at time $t > 0$ of the number of social contacts of the population of susceptible, infected and recovered individuals, respectively. The distribution of contacts of the whole population is then recovered as the sum of the three distributions

$$\begin{aligned} f(x,t)=f_S(x,t)+f_I(x,t)+f_R(x,t). \end{aligned}$$

We do not consider for simplicity of presentation disease related mortality as well as the presence of asymptomatic individuals which we aim to insert in future investigations. Therefore, we can fix the total distribution of social contacts to be a probability density for all times $t \ge 0$

$$\begin{aligned} \int _{{\mathbb {R}}_+} f(x,t)\,dx = 1. \end{aligned}$$

As a consequence, the quantities

1 $$\begin{aligned} J(t)=\int _{{\mathbb {R}}^+}f_J(x,t)\,dx,\quad J \in \{S,I,R\} \end{aligned}$$

denote the fractions, at time $t \ge 0$, of susceptible, infected and recovered respectively. For a given constant $\alpha >0$, and time $t \ge 0$, we also denote with $x_\alpha (t)$ the moment of the distribution of the number of contacts *f*(*x*, *t*) of order $\alpha$

$$\begin{aligned} x_\alpha (t) =\int _{{\mathbb {R}}^+}x^\alpha \, f(x,t)\,dx. \end{aligned}$$

In the same way, we denote with $x_{J,\alpha }(t)$ the local moments of order $\alpha$ for the distributions of the number of contacts in each class conveniently divided by the mass of the class

2 $$\begin{aligned} x_{J,\alpha }(t)= \frac{1}{{J(t)}}\int _{{\mathbb {R}}^+}x^\alpha f_J(x,t)\,dx, \quad J \in \{S,I,R\}. \end{aligned}$$

Unambiguously, we will indicate the mean and the local mean values, corresponding to $\alpha =1$, by *x*(*t*) and, respectively, $x_J(t)$, $J \in \{ S,I,R\}$.

In what follows, we assume that the various classes in the model could act differently in the social process constituting the contact dynamics. The kinetic model then follows combining the epidemic process with the contact dynamics. This gives the system

3 $$\begin{aligned} \frac{\partial f_S(x,t)}{\partial t}= & {} -K_\epsilon (f_S,f_I)(x,t) + Q_S(f_S)(x,t) \nonumber \\ \frac{\partial f_I(x,t)}{\partial t}= & {} K_\epsilon (f_S,f_I)(x,t) - \gamma _\epsilon f_I(x,t) + Q_I(f_I)(x,t) \nonumber \\ \frac{\partial f_R(x,t)}{\partial t}= & {} \gamma _\epsilon f_I(x,t) + Q_R(f_R)(x,t) \end{aligned}$$

where $\gamma _\epsilon$ is the constant recovery rate while the transmission of the infection is governed by the function $K_\epsilon (f_S,f_I)$, the local incidence rate, expressed by

4 $$\begin{aligned} {K_\epsilon (f_S,f_I)(x, t) = f_S(x,t) \int _{{\mathbb {R}}^+} \kappa _\epsilon (x,y)f_I(y,t) \,dy.} \end{aligned}$$

In full generality, we will assume that both the recovery rate $\gamma$ and the contact function $\kappa$ depend on a small positive parameter $\epsilon \ll 1$ which measures their intensity. In (4) the contact function $\kappa _\epsilon (x,y)$ is a nonnegative function growing with respect to the number of contacts *x* and *y* of the populations of susceptible and infected, and such that $\kappa _\epsilon (x, 0) = 0$. A leading example for $\kappa _\epsilon (x,y)$ is obtained by choosing

5 $$\begin{aligned} \kappa _\epsilon (x,y) = \beta _\epsilon \, x^\alpha y^\alpha , \end{aligned}$$

where $\alpha , \beta _\epsilon$ are positive constants, that is by taking the incidence rate dependent on the product of the number of contacts of susceptible and infected people. When $\alpha =1$, for example, the incidence rate takes the simpler form

6 $$\begin{aligned} {K_\epsilon (f_S,f_I)(x,t) = \beta _\epsilon \, x f_S(x,t) x_I(t)\,I(t).} \end{aligned}$$

Let us observe that with the choices done, the spreading of the epidemic depends heavily on the function $\kappa _\epsilon (\cdot ,\cdot )$ used to quantify the rate of possible contagion in terms of the number of social contacts between the classes of susceptible and infected.

In our combined epidemic contact model (3), the operators $Q_J$, $J\in \{S,I,R\}$ characterize the thermalization of the distribution of social contacts in the various classes. To that aim, observe that the evolution of the mass fractions *J*(*t*), $J\in \{S,I,R\}$ obeys to a classical SIR model by choosing $Q_S \equiv 0$ and $\kappa _\epsilon (x,y) \equiv \beta >0$, thus considering the spreading of the disease independent of the intensity of social contacts.

The $Q_J$, $J\in \{S,I,R\}$ are integral operators that modify the distribution of contacts $f_J(x,t)$, $J\in \{S,I,R\}$ through repeated interactions among individuals (Dimarco and Toscani 2019; Toscani 2020). Their action on observable quantities $\varphi (x)$ is given by

7 $$\begin{aligned} \int _{{\mathbb {R}}_+}\varphi (x)\,Q_J(f_J)(x,t)\,dx = \,\, \Big \langle \int _{{\mathbb {R}}_+}B(x) \bigl ( \varphi (x_J^*)-\varphi (x) \bigr ) f_J(x,t) \,dx \Big \rangle . \end{aligned}$$

where *B*(*x*) measures the interaction frequency, $\langle \cdot \rangle$ denotes mathematical expectation with respect to a random quantity, and $x_J^*$ denotes the updated value of the number *x* of social contacts of the *J*-th population as a result of an interaction. We discuss in the sequel the construction of the social contact model.

### On the distribution of social contacts

The process of formation of the distribution of social contacts is obtained by taking into account the typical aspects of human behaviour, in particular the search, in absence of epidemics, of opportunities for socialization. In addition to that, social contacts are due to the common use of public transportations to reach schools, offices and, in general, places of work as well as to basic needs of interactions due to work duties. As shown in Béraud (2015), this leads individuals to stabilize on a characteristic number of daily contacts depending on the social habits of a country. This quantity is represented in the following by a suitable value $\bar{x}_M$, which can be considered as the mean number of contacts relative to the population under investigation. This kind of dynamics and the relative distribution of average daily contacts observed in (Béraud 2015) is the one we aim to explain and reproduce in our model.

As a final result of our investigation, we look to a characterization of the distribution of social contacts in a multi-agent system, the so-called macroscopic behavior. This can be obtained starting from some universal assumption about the personal behavior of the single agents, i.e. from the microscopic behavior. Indeed, as in many other human activities, the daily amount of social contacts is the result of a repeated upgrading based on well-established rules. To this extent, it is enough to recall that the daily life of each person is based on a certain number of activities, and each activity carries a certain number of contacts. Moreover, for any given activity, the number of social contacts varies in consequence of the personal choice. The typical example is the use or not of public transportations to reach the place of work or the social attitudes which scales with the age. Clearly, independently of the personal choices or needs, the number of daily social contacts contains a certain amount of randomness, that has to be taken into account. Also, while it is very easy to reach a high number of social contacts attending crowded places for need or will, going below a given threshold is very difficult, since various contacts are forced by working or school activities, among others causes. This asymmetry between growth and decrease, as exhaustively discussed in (Dimarco and Toscani 2019; Gualandi and Toscani 2019), can be suitably modeled by resorting to a so-called *value function* (Kahneman and Tversky 1979) description of the elementary variation of the *x* variable measuring the average number of daily contacts. We will come back to the definition of the value function later in the section.

#### Remark 1

It is important to outline that, in presence of an epidemic, the characteristic mean number of daily contacts ${\bar{x}}_M$ reasonably changes in time, even in absence of an external lockdown intervention, in reason of the perception of danger linked to social contacts. Consequently, even if not always explicitly indicated, we will assume ${\bar{x}}_M= {\bar{x}}_M(t)$.

Furthermore, an important aspect of the formation of the number of social contacts is that their frequency is not uniform with respect to the values of *x*. Indeed, it is reasonable to assume that the frequency of interactions is inversely proportional to the number of contacts *x*. This relationship takes into account that is highly probable to have at least some contacts, and also the rare situation in which one reaches a very high values of contacts $x\gg \bar{x}_M$. On this subject, we mention a related approach discussed in Furioli et al. (2020). The introduction of a variable kernel *B*(*x*) into the kinetic equation does not modify the shape of the equilibrium density as shown later, but it allows a better physical description of the phenomenon under study, including an exponential rate of relaxation to equilibrium for the underlying Fokker-Planck type equation derived from the kinetic equation that it we will introduced next in 2.2.

Following (Dimarco and Toscani 2019; Gualandi and Toscani 2019; Toscani 2020), we will now illustrate the mathematical formulation of the previously discussed behavior. In full generality, we assume that individuals in different classes can have a different mean number of contacts. Then, the microscopic updates of social contacts of individuals in the class $J\in \{S,I,R\}$ will be taken of the form

8 $$\begin{aligned} x_J^* = x - \Phi ^\epsilon (x/{\bar{x}}_J) x + \eta _\epsilon x. \end{aligned}$$

In a single update (interaction), the number *x* of contacts can be modified for two reasons, expressed by two terms, both proportional to the value *x*. In the first one, the coefficient $\Phi ^\epsilon (\cdot )$, which takes both positive and negative values, characterizes the typical and predictable variation of the social contacts of agents, namely the personal social behavior of agents. The second term takes into account a certain amount of unpredictability in the process. A frequent choice in this setting consists in assuming that the random variable $\eta _\epsilon$ is of zero mean and bounded variance of order $\epsilon >0$, expressed by $\langle \eta _\epsilon \rangle =0$, $\langle \eta _\epsilon ^2 \rangle = \epsilon \lambda$, with $\lambda >0$. Furthermore, we assume that $\eta _\epsilon$ has finite moments up to order three.

The function $\Phi ^\epsilon$ plays the role of the *value function* in the prospect theory of Kahneman and Tversky (Kahneman and Tversky 1979, 2000), and contains the mathematical details of the expected human behavior in the phenomenon under consideration. In particular, the main hypothesis on which this function is built is that, in relationship with the mean value ${\bar{x}}_J$, $J\in \{S,I,R\}$, it is considered normally easier to increase the value of *x* (individuals look for larger networks) than to decrease it (people maintain as much connections as possible). In terms of the variable $s = x/{\bar{x}}_J$ we consider then as in (Dimarco and Toscani 2019) the class of value functions obeying to the above general rule given by

9 $$\begin{aligned} \Phi _\delta ^\epsilon (s) = \mu \frac{e^{\epsilon (s^\delta -1)/\delta }-1}{e^{\epsilon (s^\delta -1)/\delta }+1 } , \quad s \ge 0, \end{aligned}$$

where the value $\mu$ denotes the maximal amount of variation of *x* that agents will be able to obtain in a single interaction, $0 < \delta \le 1$ is a suitable constant characterizing the intensity of the individual behavior, while $\epsilon >0$ is related to the intensity of the interaction. Hence, the choice $\epsilon \ll 1$ corresponds to small variations of the mean difference $\langle x_J^* -x\rangle$. Thus, if both effects, randomness and adaptation are scaled with this interaction intensity $\epsilon$, it is possible to equilibrate their effects as we will show in Section 2.2 and obtain a stationary distribution of contacts. Note also that the value function $\Phi _\delta ^\epsilon (s)$ is such that

$$\begin{aligned} -\mu \le \Phi _\delta ^\epsilon (s) \le \mu \end{aligned}$$

and clearly, the choice $\mu <1$ implies that, in absence of randomness, the value of $x_J^*$ remains positive if *x* is positive.

Once the *elementary interaction* (8) is given, for any choice of the value function, the study of the time-evolution of the distribution of the number *x* of social contacts follows by resorting to kinetic collision-like approaches (Cercignani 1988; Pareschi and Toscani 2014), that quantify at any given time the variation of the density of the contact variable *x* in terms of the interaction operators.

Thus, for a given density $f_J(x,t)$, $J\in \{S,I,R\}$, we can measure the action on the density of the interaction operators $Q_J(f)(x,t)$ in equations (3) fruitfully written in weak form. This form corresponds to say that for all smooth functions $\varphi (x)$ (the observable quantities) we have

10 $$\begin{aligned} \dfrac{d}{dt} \int _{{\mathbb {R}}_+}\varphi (x)f_J(x,t)\,dx = \Big \langle \int _{{\mathbb {R}}_+}B(x) \bigl ( \varphi (x_J^*)-\varphi (x) \bigr ) f_J(x,t) \,dx \Big \rangle . \end{aligned}$$

Here, the expectation $$\langle \cdot \rangle $$ takes into account the presence of the random parameter $$\eta _\epsilon $$ in the microscopic interaction (8) while the function *B*(*x*), as already discussed, measures the interaction frequency. The right-hand side of equation (10) quantifies the variation in density, at a given time $$t>0$$, of individuals in the class $$J\in \{S,I,R\}$$ that modify their value from *x* to $$x_J^* $$ (loss term with negative sign) and agents that change their value from $$x_J^*$$ to *x* (gain term with positive sign). In many situations, the interaction kernel *B*(*x*) can been assumed constant (Dimarco and Toscani 2019). This simplifying hypothesis is not always well justified from a modeling point of view and thus in this work, we consider instead a non constant collision kernel *B*(*x*) (see (Furioli et al. 2020) for a discussion on this aspect). Thus, following the approach in (Furioli et al. 2020; Toscani 2020), we express the mathematical form of the kernel *B*(*x*) by assuming that the frequency of changes in the number of social contacts depends on *x* itself through the following law

$$\begin{aligned} B(x) = x^{-b}, \end{aligned}$$

for some constant $b >0$. This kernel assigns a low probability to interactions in which individuals have already a large number of contacts and assigns a high probability to interactions when the value of the variable *x* is small. The constant *b* can be suitably chosen by resorting to the following argument (Toscani 2020). For small values of the *x* variable, the rate of variation of the value function (9) is given by

11 $$\begin{aligned} \frac{d}{dx} \Phi _\delta ^\epsilon \left( \frac{x}{{\bar{x}}_J}\right) \approx {\mu \epsilon }\, {{\bar{x}}_J^{-\delta }}\, x^{\delta -1}. \end{aligned}$$

Hence, for small values of *x*, the mean individual rate predicted by the value function is proportional to $x^{\delta -1}$. Then, the choice $b = \delta$ would correspond to a collective rate of variation of the system independent of the parameter $\delta$ which instead characterizes the individual rate of variation of the value function.

In the next section, we investigate the steady states of the interaction operators $Q_J(f)(x,t), \ J\in \{S,I,R\}$ which permit to derive the macroscopic epidemic model containing the effects of social interactions among individuals described in Section 3.

### Asymptotic scaling and steady states

Let us focus on the sole social contact dynamic and introduce a time scaling

$$\begin{aligned} \tau = \epsilon t, \qquad f_{J,\epsilon }(x,\tau ) = {f_J(x,t)}, \qquad J \in \{S,I,R\}. \end{aligned}$$

which, in the following, will permit to separate the scale of the epidemic from the time scale at which, by hypothesis, the social contacts acts. Then, as a result of the scaling, the distribution $f_{J,\epsilon }$ is solution to

12 $$\begin{aligned} \dfrac{d}{d\tau } \int _{{\mathbb {R}}_+} \varphi (x) f_{J,\epsilon }(x,\tau )dx = \dfrac{1}{\epsilon } \Big \langle \int _{{\mathbb {R}}_+}B(x) \bigl ( \varphi (x_J^*)-\varphi (x) \bigr ) f_{J,\epsilon }(x,\tau ) \,dx \Big \rangle . \end{aligned}$$

We concentrate now on the analysis of the asymptotic states of the social contact dynamics in the case in which elementary interactions (8) produce extremely small modification of the number of social contacts. To that aim, note that, from the definition of $\Phi _\delta ^\epsilon$ in (9) and the assumptions on the noise term $\eta _\epsilon$ we have

13 $$\begin{aligned} \lim _{\epsilon \rightarrow 0} \frac{1}{\epsilon }{ \Phi _\delta ^\epsilon \left( \frac{x}{\bar{x}_J}\right) } = \frac{\mu }{2\delta } \left[ \left( \frac{x}{{\bar{x}}_J} \right) ^\delta -1\right] , \quad \lim _{\epsilon \rightarrow 0} \frac{1}{\epsilon }\langle \eta _\epsilon ^2\rangle = \lambda . \end{aligned}$$

Consequently, the actions of both the value function and the random part of the elementary interaction in (8) survive in the limit $\epsilon \rightarrow 0$. Observe that the limit procedure induced by (13) corresponds precisely to the situation of small interactions while, at the same time, the time scale of the dynamics is suitably scaled to see their effects. In kinetic theory, this is a well-known procedure with the name of *grazing limit*, we point the interested reader to Cordier et al. (2005); Furioli et al. (2017); Pareschi and Toscani (2014) for further details. Since if $\epsilon \ll 1$ the difference $x^*_J - x$ is small and, assuming $\varphi \in {\mathcal {C}}_0$, we can perform the following Taylor expansion

$$\begin{aligned} \varphi (x^*_J)-\varphi (x) = (x^*_J - x) \varphi '(x) + \dfrac{1}{2} (x^*_J-x)^2 \varphi ''(x) + \dfrac{1}{6}(x^*_J - x)^3\varphi '''({\hat{x}}_J), \end{aligned}$$

being ${\hat{x}}_J \in (\min \{x,x^*_J\},\max \{x,x^*_J\})$. Writing $x^*_J-x = - \Phi ^\epsilon _\delta (x/{{\bar{x}}_J})x+x\eta _\epsilon$ from (8) and plugging the above expansion in the kinetic model (12) we have for $J\in \{S,I,R\}$

$$\begin{aligned} \begin{aligned}&\dfrac{d}{d\tau } \int _{{\mathbb {R}}_+}\varphi (x) f_{J,\epsilon }(x,\tau )dx = \\&\qquad \dfrac{1}{\epsilon } \left[ \int _{{\mathbb {R}}_+} -\Phi _\delta ^\epsilon (x/{\bar{x}_J})x^{1-\delta } \varphi '(x)f_{J,\epsilon }(x,\tau )dx + \dfrac{\lambda \epsilon }{2} \int _{\mathbb R_+} \varphi ''(x)x^{2-\delta }f_{J,\epsilon }(x,\tau )dx \right] \\&\qquad + R_\varphi (f_{J,\epsilon }), \end{aligned} \end{aligned}$$

where $R_\varphi (f_{J,\epsilon })$ is the remainder

$$\begin{aligned} \begin{aligned} R_\varphi (f_{J,\epsilon })(x,\tau ) =&\dfrac{1}{2\epsilon }\int _{{\mathbb {R}}_+}\varphi ''(x) x^{-\delta }(\Phi _\delta ^\epsilon (x/{\bar{x}_J})x)^2f_{J,\epsilon }(x,t)dx \\&\dfrac{1}{6\epsilon } \left\langle \int _{{\mathbb {R}}_+} \varphi '''({\hat{x}}_J) x^{-\delta }(-\Phi ^\epsilon _\delta (x/{\bar{x}_J})x + x\eta _\epsilon )^3 f_{J,\epsilon }(x,t)dx \right\rangle . \end{aligned} \end{aligned}$$

Since, by assumption, $\varphi$ and its derivatives are bounded in ${\mathbb {R}}_+$ and decreasing at infinity and since $\eta _\epsilon$ has bounded moment of order three, namely $\langle |\eta _\epsilon |^3\rangle <+\infty$, using the bound (13) we can easily argue that in the limit $\epsilon \rightarrow 0^+$ we have

$$\begin{aligned} |R_\varphi (f_J)| \rightarrow 0. \end{aligned}$$

Hence, it can be shown that $f_{J,\epsilon }$ converges, up to subsequences, to a distribution function $f_J$ solution to

$$\begin{aligned} \dfrac{d}{d\tau } \int _{{\mathbb {R}}_+}\varphi (x)f_{J}(x,\tau )dx= & {} \int _{{\mathbb {R}}_+} \left\{ -\varphi '(x) \, \frac{\mu \,x^{1-\delta }}{2\delta }\left[ \left( \frac{x}{{\bar{x}}_J} \right) ^\delta -1\right] + \frac{\lambda }{2}\varphi ''(x)\,x^{2-\delta } \right\} \\&f_{J}(x,\tau )\,dx \end{aligned}$$

If we also impose at $x=0$ the following no-flux boundary conditions

14 $$\begin{aligned} \frac{\partial }{\partial x} (x^{2-\delta } f_J(x,\tau ))\Big |_{x=0} = 0 \quad J\in \{S,I,R\}, \end{aligned}$$

the limit equation coincides with the Fokker-Planck type equation

$$\begin{aligned} \dfrac{\partial }{\partial \tau }f_J(x,\tau ) = Q^\delta _J(f_J)(x,\tau ), \end{aligned}$$

where

15 $$\begin{aligned} Q_J^\delta (f_J)(x,\tau )= & {} \frac{\mu }{2\delta }\frac{\partial }{\partial x}\left\{ \,x^{1-\delta }\left[ \left( \frac{x}{{\bar{x}}_J} \right) ^\delta -1\right] f_{J}(x,\tau )\right\} +\frac{\lambda }{2} \frac{\partial ^2}{\partial x^2} (x^{2-\delta } f_J(x,\tau )),\nonumber \\&J \in \{S,I,R\} \end{aligned}$$

is characterized by a variable diffusion coefficient.

Remarkably enough, we can compute explicitly the equilibrium distribution of the surrogate Fokker-Planck model. Indeed, assuming that the mass of the initial distribution is one and the mean values ${\bar{x}}_J$, $J \in \{S,I,R\}$ are constant, and by setting $\nu = \mu /\lambda$, the equilibria are given by the functions

16 $$\begin{aligned} f_J^\infty (x) = C_J({\bar{x}}_J,\delta ,\nu ) x^{\nu /\delta +\delta -2} \exp \left\{ - \frac{\nu }{\delta ^2} \left( \frac{x}{{\bar{x}}_J} \right) ^\delta \right\} ,\qquad J \in \{S,I,R\}, \end{aligned}$$

where $C_J> 0$ is a normalization constant. We may rewrite the obtained steady state (16) as a generalized Gamma probability density $f_\infty (x;\theta , \chi ,\delta )$ defined by Lienhard and Meyer (1967); Stacy (1962)

17 $$\begin{aligned} f_{J,\infty }(x;\theta , \chi ,\delta ) = \frac{\delta }{\theta ^\chi } \frac{1}{\Gamma \left( \chi /\delta \right) } x^{\chi -1} \exp \left\{ - \left( x/\theta \right) ^\delta \right\} , \end{aligned}$$

characterized in terms of the shape $\chi >0$, the scale parameter $\theta >0$, and the exponent $\delta >0$ that in the present situation are given by

18 $$\begin{aligned} \chi = \frac{\nu }{\delta }+\delta -1, \quad \theta = {\bar{x}}_J \left( \frac{\delta ^2}{\nu }\right) ^{1/\delta }. \end{aligned}$$

It has to be remarked that the shape $\chi$ is positive, only if the constant $\nu = \mu /\lambda$ satisfies the bound

19 $$\begin{aligned} \nu >\delta (1-\delta ). \end{aligned}$$

Note that condition (19) holds, independently of $\delta$, when $\mu \ge \frac{\lambda }{4}$, namely when the variance of the random variation in (8) is small with respect to the maximal variation of the value function. Note moreover that for all values $\delta >0$ the moments are expressed in terms of the parameters denoting respectively the mean ${\bar{x}}_J$, $J \in \{S,I,R\}$, the variance $\lambda$ of the random effects and the values $\delta$ and $\mu$ characterizing the value function $\phi _\delta ^\epsilon$ defined in (9). Finally, the standard Gamma and Weibull distributions are included in (16), and are obtained by choosing $\delta =1$ and, respectively $\delta = \chi$. In both cases, the shape $\chi =\nu$, and no conditions are required for its positivity. It is important to notice that the function (17) expressing the equilibrium distribution of the daily social contacts in a society is in agreement with the one observed in (Béraud 2015). This was one of the goals of our investigation.

## The macroscopic social-SIR dynamics

Once we have obtained the characterization of the equilibrium distribution of the transition operators $Q_J$, $J \in \{S,I,R\}$, we are ready to study the complete system (3). To that aim, the scope of the rest of this section is the determination of the observable macroscopic equations of the introduced kinetic model.

### Derivation of moment based systems

The assumption that the dynamics leading to the contact formation is much faster than the epidemic dynamics corresponds to consider $\beta _\epsilon = \epsilon \beta$ in the formula below (4) and $\gamma _\epsilon = \epsilon \gamma$ with $\beta ,\gamma >0$, being $\epsilon \ll 1$ the scaling parameter introduced in the previous section. After introducing the scaled distributions ${f_{J}(x,\tau )}$, noticing that $\frac{\partial }{\partial \tau } f_J = \frac{1}{\epsilon }\frac{\partial }{\partial t }f_J$, we can rewrite system (3) as follows

20 $$\begin{aligned}&\frac{\partial f_S(x,\tau )}{\partial \tau } = -K(f_S,f_I)(x,\tau ) + \frac{1}{\epsilon }\, Q_S^\delta (f_S)(x,\tau ), \nonumber \\&\frac{\partial f_I(x,\tau )}{\partial \tau } = K(f_S,f_I)(x,\tau ) - \gamma f_I(x,\tau ) + \frac{1}{\epsilon }\, Q_I^\delta (f_I)(x,\tau ), \nonumber \\&\frac{\partial f_R(x,\tau )}{\partial \tau } = \gamma f_I(x,\tau ) + \frac{1}{\epsilon }\, Q_R^\delta (f_R)(x,\tau ). \end{aligned}$$

The system (20) is complemented with the boundary conditions (14) at $x=0$ and it contains all the information on the spreading of the epidemic in terms of the distribution of social contacts. Indeed, the knowledge of the densities $f_J(x,t)$, $J\in \{S,I,R\}$, allows to evaluate by integrations all moments of interest. Due to the incidence rate $K(f_S,f_I)$, as given in (4), the time evolution of the moments of the distribution functions is not explicitly computable, since the evolution of a moment of a certain order depends on the knowledge of higher order moments, thus producing a hierarchy of equations, like in classical kinetic theory of rarefied gases (Cercignani 1988).

Before discussing the closure, i.e. how to obtain a closed set of macroscopic equations, we highlight that is not restrictive to assume $\delta = 1$ since the obtained Gamma densities depend on 2 shape parameters. This choice gives for $J\in \{S,I,R\}$

21 $$\begin{aligned} Q_J^1(f_J)(x,\tau )= \frac{\mu }{2}\frac{\partial }{\partial x}\left[ \,\left( \frac{x}{{\bar{x}}_J} -1\right) f_J(x,\tau )\right] +\frac{\lambda }{2} \frac{\partial ^2}{\partial x^2} (x f_J(x,\tau )). \end{aligned}$$

In this case (18) implies $\chi = \nu$ and $\theta = \bar{x}_J/\nu$, and the steady states of unit mass, for $J\in \{S,I,R\}$, are the Gamma densities

22 $$\begin{aligned} f_J^\infty (x;\theta , \nu ) = \left( \frac{\nu }{{\bar{x}}_J}\right) ^\nu \frac{1}{\Gamma \left( \nu \right) } x^{\nu -1} \exp \left\{ -\frac{\nu }{{\bar{x}}_J}\, x\right\} . \end{aligned}$$

With this particular choice, the mean values and the energies of the densities (22), $J\in \{S,I,R\}$, are given by

23 $$\begin{aligned} \int _{{\mathbb {R}}^+} x\, f_J^\infty (x;\theta , \nu ) \, dx = {\bar{x}}_J; \quad \int _{{\mathbb {R}}^+} x^2 \, f_J^\infty (x;\theta , \nu ) \, dx =\frac{\nu +1}{\nu }{\bar{x}}_J^2. \end{aligned}$$

Following the observations of Remark 1 we can now assume ${\bar{x}}_J= x_J(t)$ where this time dependent value can be different depending on the class to which agents belong. In order to obtain the time evolution of the macroscopic observable quantities like densities and local means from the kinetic model (20), we now consider the Fokker–Planck operator (21) This operator vanishes in correspondence to a time-dependent Gamma density equilibrium with mean $x_J(t)$. With these notations, system (20) with $\delta =1$ reads then

24 $$\begin{aligned}&\frac{\partial f_S(x,\tau )}{\partial \tau } = - \beta x \,f_S(x,\tau ) x_I(t) \,I(\tau ) + \frac{1}{\epsilon }\, Q_S^1(f_S)(x,\tau ), \nonumber \\&\frac{\partial f_I(x,\tau )}{\partial \tau } = \beta x\, f_S(x,\tau ) x_I(t) \,I(\tau ) - \gamma f_I(x,\tau ) + \frac{1}{\epsilon }\, Q_I^1(f_I)(x,\tau ), \nonumber \\&\frac{\partial f_R(x,t)}{\partial \tau } = \gamma f_I(x,\tau ) + \frac{1}{\epsilon }\, Q^1_R(f_R)(x,\tau ). \end{aligned}$$

Integrating both sides of equations in (24) with respect to *x*, and recalling that, in presence of boundary conditions of type (14) the Fokker-Planck type operators are mass and momentum preserving, we obtain the system for the evolution of the fractions *J* defined in (1), $J\in \{S,I,R\}$

25 $$\begin{aligned}&\frac{d S(t)}{d t} = -\beta \, x_S(t) x_I(t) S(t)I(t), \nonumber \\&\frac{d I(t)}{d t} = \beta \, x_S(t) x_I(t) S(t)I(t) - \gamma I(t), \nonumber \\&\frac{d R(t)}{d t} = \gamma I(t), \end{aligned}$$

where we have restored the macroscopic time variable $t \ge 0$. As anticipated, unlike the classical SIR model, system (25) is not closed, since the evolution of the mass fractions *J*(*t*), $J \in \{S,I,R\}$, depends on the evolution of the local mean values $x_J(t)$. The closure of system (25) can be obtained by resorting, at least formally, to a limit procedure. In fact, as outlined in the Introduction, the typical time scale involved in the social contact dynamics is $\epsilon \ll 1$ which identifies a faster adaptation of individuals to social contacts with respect to the evolution time of the epidemic disease. Consequently, the choice of the value $\epsilon \ll 1$ pushes the distribution function $f_J(x,t)$, $J\in \{S,I,R\}$ towards the Gamma equilibrium density with a mass fraction *S*(*t*), respectively *I*(*t*) and *R*(*t*), and local mean value $x_S(t)$, respectively $x_I(t)$ and $x_R(t)$, as it can be easily verified from the differential expression of the interaction operators $Q_J^1$, $J \in \{S,I,R\}$.

Indeed, if $\epsilon \ll 1$ is sufficiently small, one can easily argue from the exponential convergence of the solution of the Fokker-Planck equation towards the equilibrium $f_S^\infty (x;\theta ,\nu )$ (see (Toscani 2020) for details), that the solution $f_S(x, t)$ remains sufficiently close to the Gamma density with mass *S*(*t*) and local mean density given by $x_S(t)$ for all times. This equilibrium distribution $f_S^\infty (x;\theta ,\nu )$ can then be plugged into the first equation of (24). Successively, by multiplying by *x* both sides of this equation (24) and integrating it with respect to the variable *x*, since the Fokker–Planck operator on the right-hand side is momentum-preserving, one obtains that the mean $x_S(t)S(t)$ satisfies the differential equation

$$\begin{aligned} \frac{d }{d t} (x_S(t)S(t)) = - \beta \, x_{S,2}(t) x_I(t) S(t)I(t), \end{aligned}$$

which depends now on the second order moment. However, it is now possible to close this expression by using the energy of the local equilibrium distribution, which can be expressed in terms of the mean value as in (23) as follows

$$\begin{aligned} x_{S,2}(t) = \frac{\nu +1}{\nu } x_S^2(t). \end{aligned}$$

Therefore, we have

$$\begin{aligned} S(t) \frac{d x_S(t)}{ d t} = - \beta \, x_{S,2}(t) x_I(t) S(t)I(t) - x_S(t)\frac{dS(t)}{d t}, \end{aligned}$$

where the time evolution of the fraction *S*(*t*) can be recovered by the first equation of system (25). Hence, the evolution of the local mean value $x_S(t)$ satisfies the equation

26 $$\begin{aligned} \frac{d x_S(t)}{d t} = - \frac{\beta }{\nu }x_{S}(t)^2 x_I(t) I(t). \end{aligned}$$

An analogous procedure can be done with the second equation in system (24), which leads to relaxation towards a Gamma density with mass fraction *I*(*t*) and local mean value given by $x_I(t)$, and with the third equation in system (24). We easily obtain in this way the system that governs the evolution of the local mean values of the social contacts of the classes of susceptible, infected and recovered individuals

27 $$\begin{aligned}&\frac{d x_S(t)}{d t} = - \frac{\beta }{\nu }x_{S}(t)^2 x_I(t) I(t), \nonumber \\&\frac{d x_I(t)}{d t} = \beta x_{S}(t) x_I(t) \left( \frac{1+\nu }{\nu } x_S(t) - x_I(t) \right) S(t), \nonumber \\&\frac{d x_R(t)}{d t} = \gamma \frac{I(t)}{R(t)}\left( x_I(t) - x_R(t)\right) . \end{aligned}$$

The closure of the kinetic system (3) around a Gamma-type equilibrium of social contacts leads then to a system of six equations for the pairs of mass fractions *J*(*t*) and local mean values $x_J(t)$, $J\in \{S,I,R\}$. In the following, we refer to the coupled systems (25) and (27) as the social SIR model (S-SIR). With respect to the classical epidemiological model from which we took inspiration, the main novelty is represented by the presence of system (27), that describes the evolution of the social contacts. It is immediate to conclude from the first equation of (27) that the local mean number of contacts of the population of susceptible individuals decreases, thus showing that the *social answer* to the presence of the pandemic is to reduce the number of social contacts. A maybe unexpected behavior is present in the second equation of (27), which indicates that, at least in the initial part of the time evolution of the S-SIR model, the class of infected individuals increases the local mean number of social contacts. This effect must be read as a consequence of the more probable transition from susceptible to infected of individuals with a high number of social contacts. A similar conclusion has been derived by resorting to a network-based model (Barthélemy et al. 2005), where it was observed that the epidemic spreads hierarchically (from highly to less highly connected nodes).

It is interesting to remark that system (27) is explicitly dependent on the positive parameter $\nu =\mu / \lambda$, which measures the heterogeneity of the population in terms of the variance of the statistical distribution of social contacts. More precisely, small values of the constant $\nu$ correspond to high values of the variance, and thus to a larger heterogeneity of the individuals with respect to social contacts. This is an important point which is widely present and studied in the epidemiological literature (Anderson and May 1991; Diekmann et al. 1990; Diekmann and Heesterbeek 2000). Concerning the COVID-19 pandemic, the influence of population heterogeneity on herd immunity has been recently quantified in (Britton et al. 2020) by testing a SEIR model on different types of populations categorized by different ages and/or different activity levels, thus expressing different levels of heterogeneity.

**Fig. 1:**
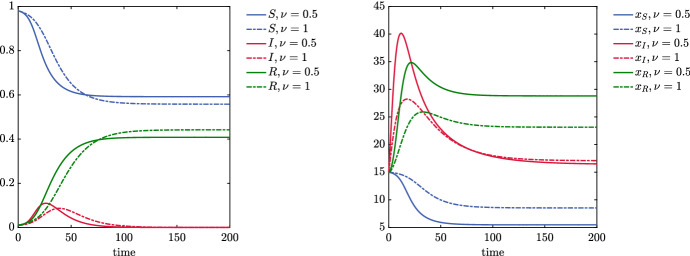
Evolution of system (25)–(27) for $\nu = 0.5$ and $\nu = 1$

A limiting case of system (27) is obtained by letting the parameter $\nu \rightarrow +\infty$, which corresponds to push the variance to zero (absence of heterogeneity). In this case, if the whole population starts with a common number of daily contacts, say ${\bar{x}}$, it is immediate to show that the number of contacts remains fixed in time, thus reducing system (25) to a classical SIR model with contact rate $\beta {\bar{x}}^2$. Hence this classical epidemiological model is contained in (25)–(27) and corresponds to consider the non realistic case of a population that, regardless of the presence of the epidemic, maintains the same fixed number of daily contacts. The described behaviors are exemplified in Fig. 1, where we considered $S(0) = 0.98$, $I(0) = R(0) = 0.01$ and mean number of contacts $x_S(0) = x_I(0) = x_R(0) = 15$ for two choices $\nu = 0.5$ and $\nu = 1$. We can easily observe how the number of recovered is affected by contact heterogeneity: the smaller the heterogeneity is, the larger the population recovers from the pandemic, we point the interested reader to (Britton et al. 2020) for an in-depth discussion on this matter.

#### Remark 2

The derivation leading to systems (25) and (27) can be easily generalized to local incidence rates (4) with a contact function of the form $\kappa (x,y) = \beta x^\alpha y^\alpha$ with $\alpha \not = 1$. Also, the procedure can be applied to equilibria which are different from the Gamma density considered here, provided this density has enough moments bounded.

#### Remark 3

The approach just described can be easily adapted to other compartmental models in epidemiology like SEIR, MSEIR (Brauer et al. 2019; Diekmann and Heesterbeek 2000; Hethcote 2000) and/or SIDHARTE (Gatto 2020; Giordano 2020). For all these cited models, the fundamental aspects of the interaction between social contacts and the spread of the infectious disease, we expect not to change in a substantial way.

### A social-SIR model with saturated incidence rate

The system (25)–(27) is a model for describing the time evolution of an epidemic in terms of the statistical distribution of social contacts, without taking into account any external intervention. However, protection measures such as lockdown strategies inevitably cause reduction in the average number of social contacts of the population which can be taken explicitly into account in our model. In the epidemiological literature, a natural way to introduce this mechanism, which dates from the work of Capasso and Serio (Capasso and Serio 1978), consists in considering a non linear incidence rate whose main feature is to be bounded with respect to the number of infected. Interestingly, on this subject, we aim to highlight that a similar behavior of the incidence rate can be directly derived starting from our social-SIR model (25)–(27). The additional hypothesis that is sufficient to introduce is that the average number $\tilde{x}_I$ of social contacts of infected is frozen as an effect, for instance, of external interventions aimed in controlling the pandemic spread. If this is the case, one can explicitly solve the first equation of system (27), which now reads

28 $$\begin{aligned} \frac{d x_S(t)}{d t} = - \frac{\beta }{\nu }x^2_{S}(t) {\tilde{x}}_I I(t), \end{aligned}$$

due to the fact that $x_I(t) = x_I(t=0)= {\tilde{x}}_I$. The exact solution of the equation (28) can then be computed and it gives

29 $$\begin{aligned} {x_S(t) = \frac{x_S(0)}{1 + \displaystyle \frac{\beta }{\nu }x_S(0) {\tilde{x}}_I \int _0^t I(s)\, ds}.} \end{aligned}$$

The above expression is a generalization of the so-called saturated incidence rate (Capasso and Serio 1978; Korobeinikov and Maini 2005) whose *classical* form is

30 $$\begin{aligned} g(I)=\frac{1}{1+\phi I} \end{aligned}$$

with $\phi$ a suitable positive constant. The same expression can be found from (25) by plugging (29) into the system. This gives

31 $$\begin{aligned}&\frac{d S(t)}{d t} = -{\bar{\beta }} \, S(t)I(t) H(I(t),t), \nonumber \\&\frac{d I(t)}{d t} = {\bar{\beta }}\, H(I(t),t) S(t)I(t) - \gamma I(t), \nonumber \\&\frac{d R(t)}{d t} = \gamma I(t), \end{aligned}$$

where $${\bar{\beta }} =\beta x_S(0){\tilde{x}}_I$$ and

32 $$\begin{aligned} {H(r(t),t) = \frac{1}{1 + \displaystyle \frac{{\bar{\beta }}}{\nu } \int _0^t r(s)\, ds}, \quad 0<r \le 1.} \end{aligned}$$

We refer in the following to this function *H*(*r*(*t*), *t*) to as the macroscopic incidence rate. Finally by approximating the integral $\int _0^t r(s)\, ds\approx t r(t)$ one obtains

33 $$\begin{aligned} H(r(t),t) = \frac{1}{1 + \displaystyle \phi (t) r(t)}, \quad 0<r \le 1. \end{aligned}$$

with $\phi (t)=({\bar{\beta }} t)/\nu$, i.e. the classical incidence rate described for the first time in (Capasso and Serio 1978).

#### Remark 4

It is possible to consider a higher influence of the number of contact in the transmission dynamics by taking $\alpha >1$ in (5). Proceeding then as described in Sect. 3.1, since

$$\begin{aligned} \int _0^{+\infty } x^\alpha f_S^\infty (x)dx = c_\alpha x_S^\alpha , \qquad c_\alpha >0, \end{aligned}$$

in the specific case $f^\infty _S$ a Gamma distribution, we would obtain

$$\begin{aligned} \dfrac{d}{dt}x_S(t) = -\dfrac{\beta }{\nu }x_S^{\alpha +1} \tilde{x}_I^\alpha I(t), \end{aligned}$$

with $x_I^\alpha (t) = {\tilde{x}}_I^\alpha >0$ whose solution is

$$\begin{aligned} x_S(t) = \dfrac{x_S(0)}{\left( 1 + \dfrac{c_\alpha \beta \alpha x_S^\alpha (0)}{\nu } {\tilde{x}}_I^\alpha \displaystyle \int _0^t I(s)ds \right) ^{1/\alpha }}. \end{aligned}$$

Therefore we obtain the closed system for the evolution of mass fractions of the type (31) which incorporates the generalized macroscopic incidence function

34 $$\begin{aligned} H(r(t),t) = \dfrac{1}{\left( 1+\phi (t)r(t)\right) ^{1/\alpha }}, \qquad 0< r(t)\le 1, \end{aligned}$$

with $$\phi (t) = c_\alpha \alpha \beta x_S^\alpha (0)t/\nu >0$$.

In the next Sect. 4 we will perform some numerical computations in which the social SIR model (31) is used in the case in which the incidence rate takes the form (32) as well as in the classical case (33) with $\phi (t)=\phi$. In a last part, we will also show that a more accurate fit of the model with the experimental data is obtained using a generalized incidence rate of the form (34).

To conclude this part, let now derive the basic reproduction number of the model introduced and discussed in the previous part. To that aim, let us note that by defining

35 $$\begin{aligned} D(S(t),I(t)) = \int _{{\mathbb {R}}^+} K(f_S,f_I)(x, t) \, dx = {\bar{\beta }} H(I(t),t)S(t)I(t). \end{aligned}$$

we have that in both cases *D*(*S*, *I*) fulfills all the properties required by the class of non-linear incidence rates considered in Korobeinikov and Maini (2005). Indeed, $D(S,0) = 0$, and the function *D*(*S*, *I*) satisfies

36 $$\begin{aligned} \frac{\partial D(S,I)}{\partial I}>0, \quad \frac{\partial D(S,I)}{\partial S} >0 \end{aligned}$$

for all $S,I >0$. Moreover *D*(*S*, *I*) is concave with respect to the variable *I*, i.e.

37 $$\begin{aligned} \frac{\partial ^2D(S,I)}{\partial I^2} \le 0, \end{aligned}$$

for all $$S,I >0$$.

Furthermore, in this case we may define classically the basic reproduction number $R_0$ of the model which is given by

38 $$\begin{aligned} R_0= \frac{1}{\gamma }\, \lim _{I\rightarrow 0, S\rightarrow 1} \frac{\partial D(S,I)}{\partial I} = \frac{{\bar{\beta }}}{\gamma }= \dfrac{\beta x_S(0){\tilde{x}}_I}{\gamma } \end{aligned}$$

**Fig. 2:**
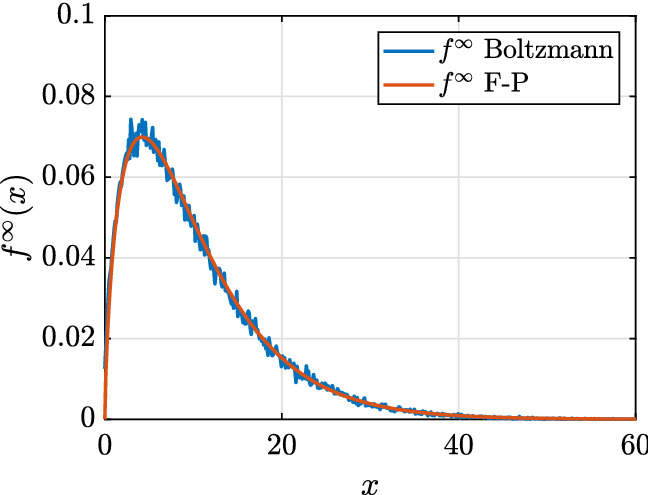
**Test 1**. Large time distribution of the Boltzmann dynamics compared with the equilibrium state of the corresponding Fokker-Planck equation. The initial distribution has been chosen of the form in (39)

## Numerical experiments

**Fig. 3:**
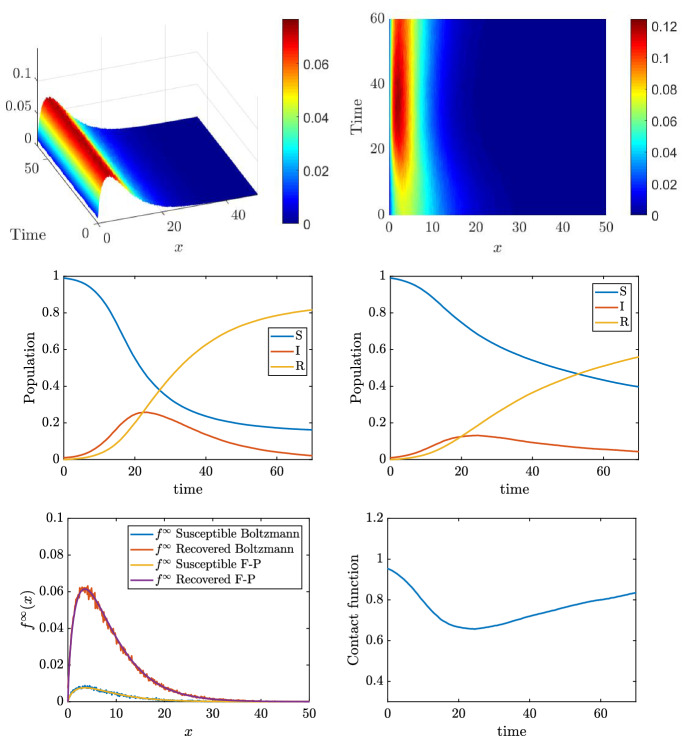
**Test 1**. Top: distribution of the daily social contacts for the two choices of the function *H*. Middle: SIR dynamics corresponding to the different choices of the different mean number of daily contact (left constant case, right as a function of the epidemic). Bottom left: final distribution of the number of contacts. Bottom right: time evolution of the contact function *H*

In addition to analytic expressions, numerical experiments allow us to visualize and quantify the effects of social contacts on the SIR dynamics used to describe the time evolution of the epidemic. More precisely, starting from a given equilibrium distribution detailing in a probability setting the daily number of contacts of the population, we show how the coupling between social behaviors and number of infected people may modify the epidemic by slowing down the number of encounters leading to infection. In a second part, we discuss how some external forcing, mimicking political choices and acting on restrictions on the mobility, may additionally improve the reduction of the epidemic trend avoiding concentration in time of people affected by the virus and consequently decreasing the probability of hospitalization peaks. In a third part, we focus on experimental data for the specific case of COVID-19 in different European countries and we extrapolate from them the main features characterizing the incidence rate *H*(*I*(*t*), *t*).

### Test 1: On the effects of the social contacts on the epidemic dynamics

We solve the social-SIR kinetic system (24). The starting point is represented by a population composed of $99.99\%$ of susceptible and $0.01\%$ of infected. The distribution of the number of contact is described by (16) with $\nu =1.65$, $\delta =1$ and $x_J=10.25$ in agreement with (Béraud 2015) while the epidemic parameters are $\beta =0.25/x_J^2$ and $\gamma =0.1$. The kinetic model (20) is solved by a splitting strategy together with a Monte Carlo approach (Pareschi and Russo 2001) where the number of samples used to described the population is fixed to $M=10^6$. The time step is fixed to $\Delta t=10^{-2}$ and the scaling parameter is $\epsilon =10^{-2}$. These choices are enough to observe the convergence of the Boltzmann dynamics to the Fokker-Planck one as shown in Fig. 2 where the analytical equilibrium distribution is plotted together with the results of the Boltzmann dynamics. For this problem, we considered a uniform initial distribution

39 $$\begin{aligned} f_0(x) = \dfrac{1}{c} \chi (x \in [0,c]), \quad c=20, \end{aligned}$$

being $\chi (\cdot )$ the indicator function. In the introduced setting, we then compare two distinct cases: in the first one we suppose that nonlinear effects in the contacts dynamics do not modify the contact rate, meaning $H(I(t),t)=1$, while the second includes the effects of the function *H*(*I*(*t*), *t*) given in (33) with $\phi (t)=\phi =10$, i.e. the classical saturated incidence rate case (Capasso and Serio 1978). The results are depicted in Fig. 3. The top right images show the time evolution of the distribution of the number of contacts for the two distinct cases, while the middle images report the corresponding evolution of the epidemic. For this second case, the function *H*(*I*(*t*), *t*) as well as the distribution of contacts for respectively the susceptible and the recovered are shown at the bottom of the same figure. We clearly observe a reduction of the peak of infected in the case in which the dynamics depends on the number of contacts with *H*(*I*(*t*), *t*) given by (33) and $\phi$ constant. We also observe a spread of the number of infected over time when sociality reduction is taken into account.

### Test 2: Forcing a change in the social attitudes

Next, we compare the effects on the spread of the disease when the population adapts its habits with a time delay with respect to the onset of the epidemic. This kind of dynamics corresponds to a modeling of a possible lockdown strategy whose effects are to reduce the mobility of the population and, correspondingly, to reduce the number of daily contacts in the population.

The setting is similar to the one introduced in Sect. 4.1 where we first consider a switch between $H=1$ to $H(I(t))=1/(1+\phi I(t))$, with $\phi =20$, when the number of infected increases. The social parameters are $\nu =1.65$, $\delta =1$ and $x_J=10.25$, as before, while the epidemic parameters are $\beta =0.25/x_J^2$ and $\gamma =0.1$, the final time is fixed to $T=70$. The initial distribution of contacts is also assumed to be of the form (39).

**Fig. 4:**
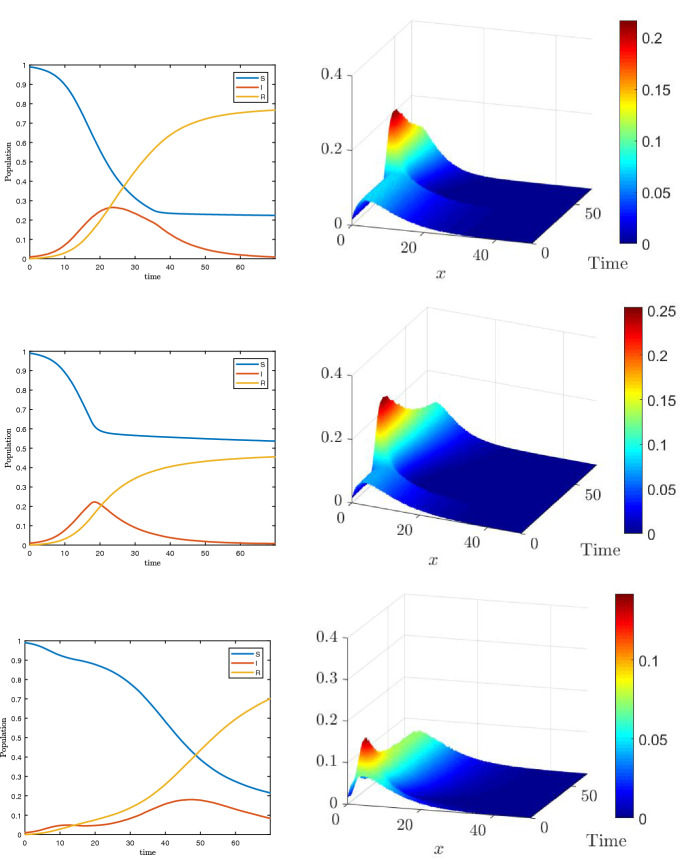
**Test 2**. Comparisons of different lockdown behaviors. Top: late lockdown. Middle: early lockdown. Bottom: early lockdown and successive relaxation

**Fig. 5:**
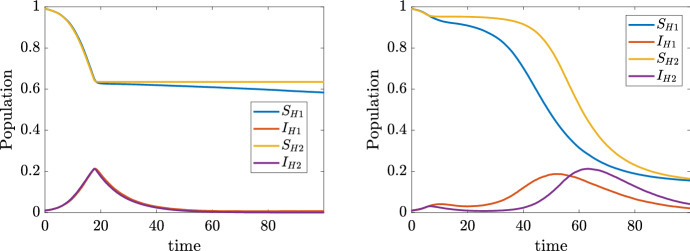
**Test 2**. Comparisons of different lockdown behaviors and different form of the incidence rate: $H_1$, $H_2$ defined in (40)–(41). Left: early lockdown. Right: early lockdown and successive relaxation

We consider three different settings, in the first one $H=1$ up to $t<35$ (days), in the second one up to $t<17$ (days) while in the third case we prescribe a lockdown for a limited amount of time ($5<t<15$) and then we relax back to $H=1$. The results are shown in Fig. 4 for both the distribution of daily contacts over time and the SIR evolution. We can consequently identify three scenarios. In the first case, on the top, we observe a slight reduction of the speed of the infection after $t>T/2$. The second case, middle images, causes a clear change to the epidemic dynamic, an inversion around $t=20$ happens. Finally, for the third case we first observe inversion and then the resurgence of the number of infected when the lockdown measures are relaxed. We consider now an alternative scenario whose results are depicted in Fig. 5. In this situation, we compare the case

40 $$\begin{aligned} H_1(I(t),t)=\dfrac{1}{1+\phi I(t)} \end{aligned}$$

with the case

41 $$\begin{aligned} H_2(I(t),t) = \dfrac{1}{1 + \phi \int _0^t I(s)\, ds}. \end{aligned}$$

The value of $\phi$ is increased to $\phi =50$ to enhance the different behaviors of the two models in two of the three possible lockdown scenarios described previously. In the case of the early lockdown (lockdown after 17 days), the difference between the two models is small. In particular, the case of the incidence rate depending on the instantaneous number of infected gives, as expected, a slightly larger number of total infected in time. The case of early lockdown followed by a relaxation exhibits much stronger differences. In this latter, a time shift between the second wave is clearly present while the incidence rate depending on the history of the pandemic gives a higher pick of infected. For this problem, the simulations are run for $T=100$ days.

### Test 3: Extrapolation of the incidence rate shape from data

In this part, we consider experimental data about the dynamics of COVID-19 in three European countries: France, Italy and Spain. For these three countries, the evolution of the disease, in terms of reported cases, evolved in rather different ways. The estimation of epidemiological parameters for compartmental-like models is an inverse problem of, in general, difficult solution for which different approaches can be considered. We mention in this direction a very recent comparison study (Liu et al. 2020). It is also worth to mention that often the data are partial and heterogeneous with respect to their assimilation, see for instance the discussions in Albi et al. (2021); Capaldi (2012); Chowell (2017); Roberts (2013). This makes the fitting problem challenging and the results naturally affected by uncertainty.

The data we employ, concerning the actual number of infected, recovered and deaths of COVID-19 are publicly available from the John Hopkins University GitHub repository (Dong et al. 2020). For the specific case of Italy, we considered instead the GitHub repository of the Italian Civil Protection Department^1^. In the following, we present the adopted fitting approach which is based on a strategy with two optimization horizons (pre-lockdown and lockdown time spans) depending on the different strategies enacted by the governments of the considered European countries.

In details, we considered first the time interval $t \in [t_0,t_\ell ]$, being $t_\ell$ the day in which lockdown started in each country (Spain, Italy and France) and $t_0$ the day in which the reported cases hit 200 units. The lower bound $t_0$ has been imposed to reduce the effects of fluctuations caused by the way in which data are measured which have a stronger impact when the number of infected is low. Once the time span has been fixed, we then considered a least square problem based on the minimization of a cost functional ${\mathcal {J}}$ which takes into account the relative $L^2$ norm of the difference between the reported number of infected and the reported total cases ${\hat{I}}(t)$, ${\hat{I}}(t)+{\hat{R}}(t)$ and the evolution of *I*(*t*) and $I(t)+R(t)$ prescribed by system (31) with $H \equiv 1$. In practice, we solved the following constrained optimisation problem

42 $$\begin{aligned} \text {min}_{\beta ,\gamma } {\mathcal {J}}({\hat{I}}, {\hat{R}}, I, R) \end{aligned}$$

where the cost functional ${\mathcal {J}}$ is a convex combination of the mentioned norms and assumes the following form

$$\begin{aligned} J({\hat{I}}, {\hat{R}}, I, R) = p \frac{ \Vert {\hat{I}}(t)-I(t)\Vert _2}{\Vert {\hat{I}}(t) \Vert _2}+ (1-p) \frac{ \Vert {\hat{I}}(t)+{\hat{R}}(t)-I(t)-R(t) \Vert _2}{\Vert {\hat{I}}(t) + {\hat{R}}(t) \Vert _2} \end{aligned}$$

We then choose $p = 0.1$ and we look for a minimum under the constraints

$$\begin{aligned} \begin{aligned} 0\le \beta \le 0.6, \qquad 0.04 \le \gamma \le 0.06. \end{aligned} \end{aligned}$$

In Table 1 we report the results of the performed parameter estimation together with the resulting reproduction number $R_0$ defined in (38).

**Table 1:** **Test 3**. Model fitting parameters in estimating the reproduction number for the COVID-19 outbreak before lockdown in various European countries

	$\underset{\text {Mar 5-Mar 17}}{\text {France}}$	$\underset{\text {Feb 24-Mar 9}}{\text {Italy}}$	$\underset{\text {Mar 5-Mar 14}}{\text {Spain}}$
$\beta$	0.300686	0.317627	0.370362
$\gamma$	0.040000	0.058391	0.043168
$R_0$	7.5172	5.4397	8.5795

Once that the contagion parameters have been estimated in the pre-lockdown time span, we successively proceeded with the estimation of the shape of the function *H* from the data. To estimate this latter quantity, we solved a second optimization problem which reads

43 $$\begin{aligned} \text {min}_H {\mathcal {J}} \end{aligned}$$

in terms of *H* where ${\mathcal {J}}$ is the same functional of the previous step and where in the evolution of the macroscopic model the values $\beta ,\gamma$ have been fixed as a result of the first optimization in the pre-lockdown period. The parameters chosen for (43) are $p = 0.1$ while the constraint is

$$\begin{aligned} \begin{aligned} 0\le H \le 1. \end{aligned} \end{aligned}$$

**Fig. 6:**
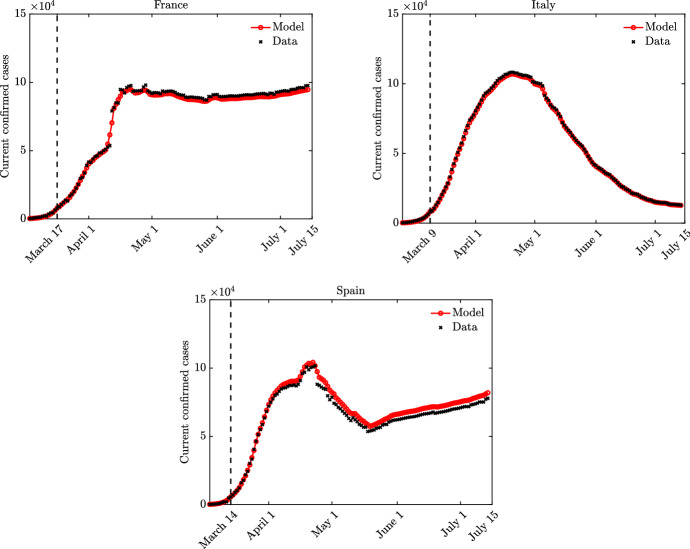
**Test 3**. Fitting of the parameters of model (31) where $\beta ,\gamma >0$ were estimated before the lockdown measures assuming $H \equiv 1$. The parameters characterizing the function $H(\cdot )$ in (31) have been computed during and after lockdown at regular interval of time, up to July 15. The lockdown measures change in each country (dashed line)

The second optimisation problem has been solved up to last available data for each country with daily time stepping $\Delta t = 1$ and over a time window of three days. This has been done with the scope of regularizing possible errors due to late reported infected and smoothing the shape of *H*. Both optimisation problems (42)–(43) have been tackled using the Matlab functions fmincon in combination with a RK4 integration method of the system of ODEs. In Fig. 6, we present the result of such fitting procedure between the model (31) and the experimental data.

**Fig. 7:**
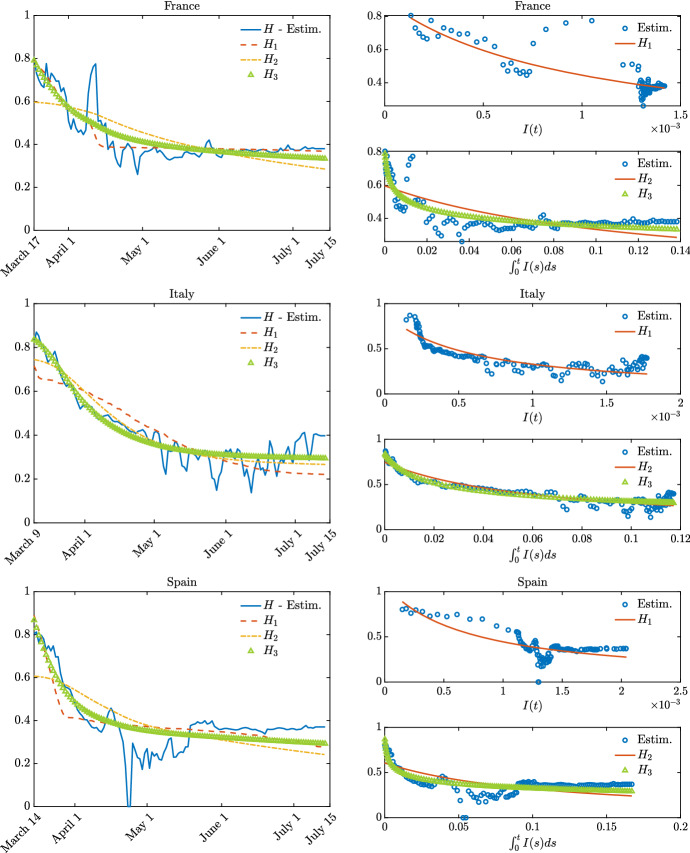
**Test 3**. Estimated shape of the function *H* in several European countries (left plots) and its dependency on the variables *I*(*t*) and $\int _{0}^{t}I (s) ds$ (right plots)

Next, we seek to understand numerically the dependencies of the function *H* from the number of infected. In particular, we first define the candidate incidence functions $H_1$, $H_2$ and $H_3$ as

$$\begin{aligned} \begin{aligned} H_1(I(t),t)&= \dfrac{c}{1 + \displaystyle \phi I(t)}, \\ H_2(I(t),t)&= \dfrac{c}{1 + \phi \int _0^t I(s)\, ds},\\ H_3(I(t),t)&= \dfrac{c}{\left( 1 + \phi \int _0^t I(s)\, ds\right) ^{1/\alpha }}, \end{aligned} \end{aligned}$$

with $c>0$, accordingly with (32)–(33) and (34) where $\phi$ and $\alpha$ are free parameters which are determined through a least square minimization approach that best fit the estimated curve with conditions $\phi >0, \alpha \ge 1$. The results of this procedure are presented in Table 2 and in Fig. 7. In Table 2, we reported the values of the fitting coefficients $\phi$ and $\alpha$ and to evaluate the goodness of fit we reported the so-called determination coefficient $R^2$, where $R^2\approx 1$ indicates perfect fit. We can observe that the optimization gives acceptable results for the different forms of the incidence function especially in the right column of Fig. 7, it appears clearly that the functions $H_2$ and $H_3$ are able to better explain the estimated values of *H* especially after the epidemic peak. In particular, the fits of the model with the available data when $H_3$ is used are particular good. This fact may indicate that people are rather fast to apply social distancing, and therefore to reduce their average number of contacts, whereas they tend to restore the pre-pandemic average contact rate more slowly, possibly due to further memory effects.

**Table 2:** Fitting parameters for the estimate of the contact functions $H_1$, $H_2$, and $H_3$ in different countries based on the evolution of *H*(*t*) solution of the optimisation problem (43). The corresponding determination coefficient $R^2$ is also reported

$H_1$	$\underset{\text {Mar 18 - Jul 15}}{\text {France}}$	$\underset{\text {Mar 10 - Jul 15}}{\text {Italy}}$	$\underset{\text {Mar 15 - Jul 15}}{\text {Spain}}$
*c*	0.8972	0.8954	1.078
$\phi$	1015	1720	1423
$R^2$	0.7515	0.7097	0.5733
$H_2$	$\underset{\text {Mar 18 - Jul 15}}{\text {France}}$	$\underset{\text {Mar 10 - Jul 15}}{\text {Italy}}$	$\underset{\text {Mar 15 - Jul 15}}{\text {Spain}}$
*c*	0.5973	0.7465	0.6076
$\phi$	7.91	15.39	9.073
$R^2$	0.496	0.8507	0.3692
$H_3$	$\underset{\text {Mar 18 - Jul 15}}{\text {France}}$	$\underset{\text {Mar 10 - Jul 15}}{\text {Italy}}$	$\underset{\text {Mar 15 - Jul 15}}{\text {Spain}}$
*c*	0.8141	0.8424	0.9066
$\phi$	1437	141.6	1624
$\alpha$	5.9312	2.7263	4.9578
$R^2$	0.7375	0.8872	0.6722

### Test 4: S-SIR model with fitted contact function

In this last part, we discuss the results of the social-SIR model when the contact function has the shape extrapolated in the previous paragraph. In particular, we aim in studying the role of the extrapolated incidence function *H* in the fitting of the model with the experimental data. Our choice consists in considering *H* dependent on the total number of infected, where however, to leave freedom to the model to produce qualitative trends that are in agreement with the data, we leave three free parameters. In details the incidence function takes the form

44 $$\begin{aligned} H(t,I(t))=\frac{c}{\left( 1+\phi \int _{0}^{t}I(s)ds\right) ^{1/\alpha }}, \end{aligned}$$

where the starting point are the parameters of Table 2 with slight modification with the scope of trying to reproduce the best fit possible with the data at disposal. In order to compare qualitatively the observed curve of infected and the theoretical one, we consider the following setting for the three countries under study: $\nu =1.65, \delta =1$ and $x_J=10.25$, $\Delta t=0.01$, $\epsilon =0.01$, and $M=10^5$ particles for the DSMC numerical approximation of the kinetic model. Moreover, we suppose $S(t=0)$ and $I(t=0)$ to match the relative number of susceptible and infected of each country at the time in which we start our comparison.

**Fig. 8:**
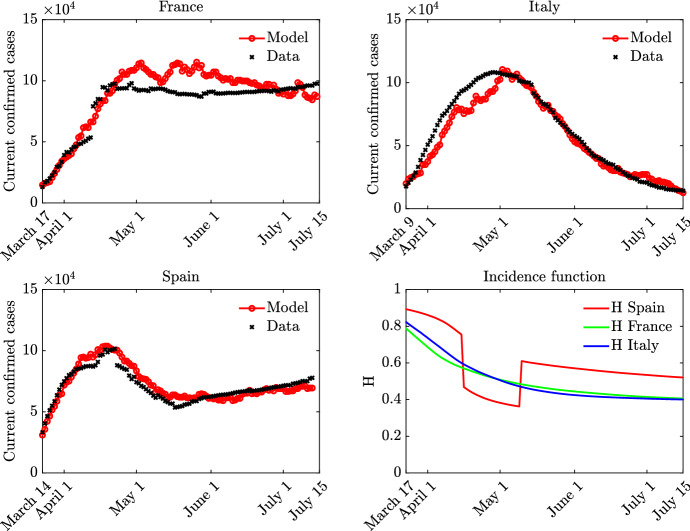
**Test 4**. Left: Number of infected over time for the S-SIR model when memory effects are taken into account in the contact function. Top left France, Top right Italy, Bottom left Spain, Bottom right effective value of the incidence function

In Fig. 8 we show the profiles of the infected over time together with the shape of the function *H* again over time. The results show that with the choices done for the incidence rate function, it is possible to reproduce, at least qualitatively, the shape of the trend of infected during the pandemic observed in Italy and in France.

It is worth to remark that the considered social parameters have been estimated only in the case of France, see (Béraud 2015), and we assumed that the initial contact distribution is the same for the Italian case.

We now focus on the case of Spain. For this country, according to Fig. 6, the trend of infected undergoes a deceleration during the lockdown period. This can be also clearly observed in Fig. 7 where the extrapolated shape of the contact function *H* is shown. Let also observe that while the global behavior of this function is captured by the fitting procedure, we however lose the minimum which takes place around end of April. This minimum is responsible of the deceleration in the number of infected and can be brought back to a strong external intervention in the lifestyle of the Spain country with the scope of reducing the hospitalizations. This effect can be reproduced by our model by imposing the same behavior in the function *H*. The Fig. 8 reports on the bottom right the shape of the function *H* over time for in particular this last case. The results show that the S-SIR model is capable to qualitatively reproduce the data.

## Conclusions

The development of strategies for mitigating the spreading of a pandemic is an important public health priority. The recent case of COVID-19 pandemic has seen as main strategy restrictive measures on the social contacts of the population, obtained by household quarantine, school or workplace closure, restrictions on travels, and, ultimately, a total lockdown. Mathematical models represent powerful tools for a better understanding of this complex landscape of intervention strategies and for a precise quantification of the relationships between potential costs and benefits of different options (Ferguson 2006). In this direction, we introduced a system of kinetic equations coupling the distribution of social contacts with the spreading of a pandemic driven by the rules of the SIR model, aiming to explicitly quantify the mitigation of the pandemic in terms of the reduction of the number of social contacts of individuals. The kinetic modeling of the statistical distribution of social contacts has been developed according to the recent results in (Béraud 2015), which present an exhaustive description of the contact dynamic in the France population, divided by categories. The characterization of the equilibrium distribution of social contacts in the form of a Gamma density allowed to obtain a new macroscopic system (25)–(27) of six differential equations giving the joint evolution of mass fractions and local mean values of daily contacts for the different classes of individuals: susceptible, infected and recovered. It is worth to notice that the obtained system (27), driving the evolution of the local mean values of social contacts, was found explicitly dependent from a parameter which can be directly linked to the variance of the equilibrium Gamma distribution. This permitted to naturally include in the set of forecasting equations a measurable effect of the heterogeneity of the social contacts. In this direction, with respect to a direct choice of a nonlinear incidence rate in the classical SIR model, as first considered in Capasso and Serio (1978), the system (25)–(27) allows for an explanation of the relation between the contacts among individuals and the spread of an epidemic. Moreover, the new presented model gives a better description of the effects of the contacts reduction policies in the spread of a virus in a population. The numerical experiments confirm that the kinetic system is able to capture most of the macroscopic phenomena related to the effects of partial lockdown strategies, and, eventually to maintain pandemic under control.

## Footnotes

1 Presidenza del Consiglio dei Ministri, https://github.com/pcmdpc/COVID-19
